# Supplementation of Water-Soluble Vitamins Reduces Hyperhomocysteinemia, Insulin Resistance, and High-Sensitivity C-reactive Protein in Prehypertension Subjects

**DOI:** 10.7759/cureus.33481

**Published:** 2023-01-07

**Authors:** Prashanth Talikoti, Zachariah Bobby, Abdoul Hamide

**Affiliations:** 1 Biochemistry, Employees' State Insurance Corporation (ESIC) Medical College, Gulbarga, IND; 2 Biochemistry, Jawaharlal Institute of Postgraduate Medical Education and Research, Puducherry, IND; 3 Medicine, Jawaharlal Institute of Postgraduate Medical Education and Research, Puducherry, IND

**Keywords:** homa-ir, water-soluble vitamins, prehypertension, c-reactive protein, insulin resistance, homocysteine

## Abstract

Background: Prehypertensives are at higher risk of developing cardiovascular diseases. Hyperhomocysteinemia, insulin resistance, and increased high-sensitivity C-reactive protein (hs-CRP) are independent risk factors for the development of cardiovascular complications. In prehypertensives, specific therapeutic approaches can be implemented at the earliest to prevent the onset of overt hypertension. So the present study was performed to study the effect of supplementation of water-soluble vitamins on cardiovascular risk factors like homocysteine, insulin resistance, and C-reactive protein in prehypertensive subjects.

Methods: Sixty prehypertensive subjects were recruited into the study based on inclusion and exclusion criteria and randomized into two groups of 30 each. One group was given a placebo and the other was given water-soluble vitamins for four months. Serum homocysteine, insulin, homeostatic model assessment of insulin resistance (HOMA-IR), and hs-CRP were assayed.

Results: After four months of treatment with water-soluble vitamins, there was a significant decrease in levels of serum homocysteine, hs-CRP, and HOMA-IR when compared to placebo treatment. After four months of treatment, there was a significant decrease in the levels of hs-CRP, homocysteine, and HOMA-IR in groups treated with water-soluble vitamins compared to the basal levels.

Conclusion: In subjects with prehypertension, supplementation of water-soluble vitamins decreases the level of homocysteine, insulin resistance, and hs-CRP.

## Introduction

The term “prehypertension” is mainly termed to define subjects with elevated risk for the development and progression of cardiovascular disease (CVD). According to the updated 2014 Eighth Joint National Committee (JNC-8), the definition of prehypertension is stated as systolic blood pressure between 120 and 139 mm of Hg and diastolic blood pressure between 80 and 89 mm of Hg, respectively [1]. Prehypertension can progress to hypertension and is also associated with metabolic syndrome, mortality, stroke, and coronary heart disease [2]. The prevalence of prehypertension in India is alarmingly high with 41.5% in the age group of 18 to 24 years, 43.8% in the age group of 25 to 44 years, and 36.3% in the age group of 45 to 69 years and it is more in males compared to females [3]. Effective therapeutic interventions at this stage may prevent or delay the onset of hypertension at prehypertensive stages. Prehypertension is associated with target organ damage such as subclinical atherosclerosis, increased thickness of tunica intima and media, renal atherosclerosis, arterial hyalinosis, and endothelial dysfunction [4]. Various publications highlight the association between total homocysteine (tHcy) levels and the development of coronary heart and vascular disease [5-7]. Hyperhomocysteinemia-mediated vascular complications are largely observed in hypertensive patients. The association between plasma homocysteine with blood pressure has been reported in earlier studies [8]. The increased C-reactive protein (CRP) is a sign of systemic inflammation. Previous studies show increased CRP levels in prehypertensive subjects with impaired arterial stiffness [9]. Further, it has been shown that insulin resistance (IR) measured in terms of a metabolic score for IR (METS-IR) has a significant association with prehypertension in normoglycemic subjects [10].

Various studies have documented decreased homocysteine, insulin resistance, and high-sensitivity CRP (hs-CRP) in patients with supplementation of water-soluble vitamins in subclinical atherosclerosis, metabolic syndrome, and in patients with myocardial infarction [11-13]. In this backdrop, the present study was conducted to evaluate the role of supplementation of water-soluble vitamins in the reduction of cardiovascular risk factors such as homocysteine, insulin resistance, and CRP in prehypertensive subjects.

## Materials and methods

The study was a single-blinded, randomized, placebo-controlled study. The study was approved by the institute's research and ethics committee. The study was registered in the Clinical Trials Registry of India (registration number: CTRI/2011/12/002250).

Inclusion criteria

Subjects with prehypertension as defined by the JNC-8, between the age group of 25 and 45 years with body mass index < 30 kg/m2 were included in the study after obtaining written informed consent.

Exclusion criteria

Patients with a history of coronary heart disease, diabetes, kidney disease, infections, smoking, alcohol intake, pregnant women, and those who had a chronic illness and were on medication were excluded from the study.

Blood pressure was recorded three times in both arms using a mercury sphygmomanometer with five minutes of rest in between and the average value was used in the study.

Study design

Sixty subjects with prehypertension in the age group of 25 to 45 years were enrolled and randomized into two groups (n = 30 each). For the period of four months, water-soluble vitamins (Becosules capsules, Pfizer Limited, Navi Mumbai, India) were given to group A, and placebo (500 mg of starch/day, in the form of two capsules of 250 mg each prepared by Acumen Pharmaceuticals, Pondicherry, India) was given to group B. The composition of Becosules is given in Table 1.

**Table 1:** The composition of each Becosules capsule

Vitamins	Composition of Becosules/capsule
Thiamine	10 mg
Riboflavin	3 mg
Cyanocobalamine	15 µg
Folic acid	1.5 mg
Riboflavin	10 mg
Niacin	100 mg
Biotin	100 µg
Calcium pantothenate	50 mg
Vitamin C	150 mg

Anthropometric measurements

Height and weight were evaluated by the standard procedure and the BMI was calculated (weight in kilograms divided by square of height in meters). The patients with BMI ≤ 30 kg/m2 were recruited for the study.

Blood collection

After overnight fasting, all subjects who were included in the study were asked to report at the urban health center of the institute, and 5 ml of blood was collected to measure the basal level of various parameters and they were informed to take two capsules per day (group A was given water-soluble vitamins and group B was given placebo) and again blood sample was collected at the end of the second and fourth month. Serum glucose was analyzed immediately after the collection of the blood and the rest of the separated serum sample was stored at -80°C for the analysis of homocysteine, vitamin B12, folate, insulin, and hs-CRP.

Analytical methods

Serum glucose was analyzed using commercial kits adapted to Olympus AU400 auto analyzer (Olympus, Center Valley, PA), and by using competitive immunoassay direct chemiluminescence technology (ADVIA Centaur, Siemens Healthineers, Erlangen, Germany). Serum homocysteine, vitamin B12, folate, insulin, and hs-CRP were assayed by enzyme-linked immunosorbent assay (ELISA) from Diagnostics Biochem Canada Inc (London, Canada). Insulin resistance was calculated by the following formula: homeostatic model assessment of insulin resistance (HOMA-IR) = fasting glucose (mmol/L) x fasting insulin (µU/mL)/22.5 [14].

Data analysis

Mean ± SD was used to represent data. The association of variables between the groups was analyzed using an independent t-test. Two-way ANOVA followed by Bonferroni's multi-comparison post hoc test was used to analyze the effect of water-soluble vitamin treatment between the groups. Statistical significance was considered if the p-value was <0.05. All analyses were performed with Graphpad Prism version 5 (GraphPad Software, San Diego, CA).

## Results

The mean age of the study participants in groups A and B was found to be 37.5 ± 6.2 and 36.3 ± 5.1 years, respectively. The BMI of group A and B subjects was 23.9 ± 21 and 24.9 ± 2.0 kg/m^2^, respectively,^ ^and the difference was found to be non-significant. The details of anthropometric data are shown in Table 2.

**Table 2:** Comparison of general variables between group A on water-soluble vitamins and group B on placebo in subjects with prehypertension (before treatment) Values are expressed as mean ± SD. NS = not significant; p < 0.01 significant.

Variables	Group A (n = 30)	Group B (n = 30)	P-value
Age (years)	37.5 ± 6.2	36.3 ± 5.1	NS
M/F	21/9	18/12	-
Weight (kg)	64.8 ± 6.0	62.8 ± 5.9	NS
BMI (kg/m^2^)	23.9 ± 2.1	24.9 ± 2.0	NS

The levels of cardiovascular risk parameters at basal, end of two months, and four months in group A prehypertensive subjects treated with water-soluble vitamins are shown in Table 3. There was a statistically significant (p < 0.05) decrease in the levels of homocysteine, hs-CRP, insulin, and HOMA-IR after two and four months of treatment when compared with basal level. With water-soluble vitamin supplementation, the levels of vitamin B12 and folate significantly (p < 0.05) increased compared to baseline levels.

**Table 3:** Effect of water-soluble vitamins on cardiovascular risk parameters at the end of two months and four months in subjects with prehypertension Values are expressed as mean ± SD. ^α^ Comparison of basal vs. two months within the group with p-value < 0.05. ^β^ Comparison of basal vs. four months within the group with p-value < 0.05. ^γ^ Comparison of two months vs. four months within the group with p-value < 0.05. The statistical analyses were carried out by two-way repeated measures ANOVA using Graphpad Prism software. hs-CRP: high-sensitivity C-reactive protein; HOMA-IR: homeostatic model assessment of insulin resistance.

Variables	Subjects on water-soluble vitamins (n = 30)		
	Basal	2 months	4 months
Homocysteine (µmol/L)	20.7 ± 7.3	13.03 ± 3.1^α^	11.64 ± 3.7^β^
Folate (nmol/L)	9.06 ± 0.68	37.84 ± 11.56^α^	38.30 ± 15.18^β^
Vitamin B12 (pmol/L)	177.4 ± 29.44	210.2 ± 35.5^α^	248.8 ± 45.7^β,γ^
hs-CRP (ng/mL)	3846 ± 2327	2765 ± 656.1^α^	2536 ± 1410^β^
Insulin (µU/mL)	14.8 ± 6.7	7.0 ± 1.1^α^	7.2 ± 2.2^β^
HOMA-IR	2.2 ± 0.8	1.7 ± 0.4^α^	1.4 ± 0.4^β^

The levels of cardiovascular risk parameters at basal, end of two months, and four months in a group of prehypertensive subjects who were on placebo are shown in Table 4. There was a significant (p < 0.05) decrease in the levels of insulin at the end of two and four months of treatment when compared to their basal levels within the group. In subjects supplemented with a placebo, there was no significant difference (p > 0.05) in homocysteine, folate, vitamin B12, hs-CRP, and HOMA-IR.

**Table 4:** Cardiovascular risk parameters at baseline, end of two months, and four months in subjects on placebo Values are expressed as mean ± SD. ^α^ Comparison of basal vs. two months within the group with p-value < 0.05. ^β^ Comparison of basal vs. four months within the group with p-value < 0.05. The statistical analyses were carried out by two-way repeated measures ANOVA using Graphpad Prism software. hs-CRP: high-sensitivity C-reactive protein; HOMA-IR: homeostatic model assessment of insulin resistance.

Variables	Subjects on placebo (n = 30)		
	Basal	2 months	4 months
Homocysteine (µmol/L)	20.6 ± 6.7	17.8 ± 6.2	20.98 ± 5.2
Folate (nmol/L)	11.78 ± 5.6	11.33 ± 2.9	14.96 ± 4.0
Vitamin B12 (pmol/L)	204.9 ± 65.3	188.8 ± 29.0	212.7 ± 53.3
hs-CRP (ng/mL)	3650 ± 876.4	3212 ± 747	3018 ± 1033
Insulin (µU/mL)	14.5 ± 1.7	12.7 ± 0.5^α^	11.8 ± 2.3^β^
HOMA-IR	2.3 ± 0.8	2.07 ± 0.6	2.3 ± 0.5

Table 5 compares the difference in levels of cardiovascular risk factors in prehypertensive subjects treated with water-soluble vitamins and placebo at the end of two and four months. There was a significant decrease in the levels of HOMA-IR, homocysteine, and hs-CRP at the end of treatment with water-soluble vitamins when compared to the effect of treatment with placebo (Table 5). Folate was significantly increased after treatment with water-soluble vitamins at the end of two and four months when compared to placebo, whereas vitamin B12 was increased significantly only at the end of four months of treatment. There was a significant reduction in insulin resistance in subjects treated with water-soluble vitamins at the end of two and four months when compared to placebo.

**Table 5:** Effect of water-soluble vitamins on cardiovascular risk parameters at the end of two months and four months in subjects with prehypertension as compared to placebo Values are expressed as mean ± SD. * Comparison of subjects on water-soluble vitamins with placebo with p-value < 0.05. The statistical analyses were carried out by two-way repeated measures ANOVA using Graph pad prism software. hs-CRP: high-sensitivity C-reactive protein; HOMA-IR: homeostatic model assessment of insulin resistance.

Biochemical variables	Subjects on water-soluble vitamins (n = 30)		Subjects on placebo (n = 30)	
	2 months	4 months	2 months	4 months
Homocysteine (µmol/L)	13.03 ± 3.1*	11.64 ± 3.7*	17.8 ± 6.2	20.98 ± 5.2
Folate (nmol/L)	37.84 ± 11.56*	38.30 ± 15.18*	11.33 ± 2.9	14.96 ± 4.0
Vitamin B12 (pmol/L)	210.2 ± 35.5	248.8 ± 45.7*	188.8 ± 29.0	212.7 ± 53.3
hs-CRP (ng/mL)	2765 ± 656.1*	2536 ± 1410*	3212 ± 747	3018 ± 1033
Insulin (µU/mL)	7.0 ± 1.1*	7.2 ± 2.2*	12.7 ± 0.5	11.8 ± 2.3
HOMA-IR	1.7 ± 0.4*	1.4 ± 0.4*	2.07 ± 0.6	2.3 ± 0.5

## Discussion

In a previous study on subjects with prehypertension, we observed that homocysteine, insulin resistance, and hs-CRP were elevated [15]. In prehypertensive subjects, we observed that malondialdehyde, a reliable marker of lipid peroxidation and oxidative stress, and blood pressure significantly decreased on supplementation of water-soluble vitamins [16]. In this backdrop, the present study delineates the efficacy of water-soluble vitamin supplementation on the elevated levels of homocysteine, insulin resistance, and hs-CRP among prehypertensive subjects in comparison with the placebo.

Blood levels of homocysteine are mediated by various intracellular metabolic reactions and among these, folic acid serves as a substrate, and vitamin B12 acts as a coenzyme [17]. The rampant generation of free radicals is the major mechanism of homocysteine-mediated endothelial dysfunction. Homocysteine contains a highly reactive free sulfhydryl group (-SH), which undergoes autoxidation to generate reactive superoxide radicals. It has been reported that nearly 98% of plasma homocysteine is in the oxidized state either in any of the two forms, i.e., homocysteine disulfide dimer or mixed disulfide, and is usually associated with serum proteins such as albumin [18]. Further, homocysteine-mediated endothelial dysfunction is also due to the reduction in nitric oxide (NO) bioavailability [19]. Oxidation of homocysteine leads to the generation of free radicals, which is in line with our previously reported study [16].

In the present study, homocysteine was significantly reduced and vitamin B12 and folate were significantly increased on supplementation with water-soluble vitamins for a period of four months in prehypertensives. In contrast, there was no significant difference in these parameters among the placebo group. A recent randomized pilot trial done by Lindschinger et al. [20] shows that supplementation with B complex vitamins significantly increased the levels of folic acid, vitamin B12, vitamin B6, and total antioxidant capacity and decreased the homocysteine levels in healthy subjects. In another study done by Setola et al. [12], supplementation of vitamin B12 and folate in metabolic syndrome patients significantly improved insulin resistance and endothelial dysfunction, along with decreasing homocysteine levels. The Women's Antioxidant and Folic Acid Cardiovascular Study conducted on women with cardiovascular risk shows that after 7.3 years of combined treatment with folic acid, vitamin B6, and vitamin B12, homocysteine concentrations were reduced by 18% [21]. Thus water-soluble vitamin supplementation may reduce cardiovascular risk, which is in line with the current study finding.

Mounting studies reveal the association between prehypertension and insulin resistance [22,23]. Previous studies show that insulin resistance followed by hyperinsulinemia increases the activity of the sympathetic nervous system [24]. This increases renal tubular sodium reabsorption, modulates cation transport, induces vascular smooth muscle cell hypertrophy, and decreases basal production of nitric oxide by the vascular endothelium leading to endothelial dysfunction [25]. These biochemical derangements cause the progression from prehypertension to hypertension [26]. In the event of insulin resistance, BH4 (tetrahydrobiopterin) synthesis is downregulated, which preludes to decreased NO synthesis and leads to vascular dysfunction in patients with type 2 diabetes mellitus [27]. The increased availability of folic acid during vitamin supplementation might stimulate BH4 formation and NO synthesis, and in turn, reduce endothelial dysfunction[28].

In the present study, in prehypertensive subjects, when compared to subjects on placebo, fasting insulin levels and insulin resistance are reduced when treated with water-soluble vitamins. The reduction was also significant when compared to their basal levels in these subjects. Setola et al. from Italy also had the same findings as ours and concluded that folate and vitamin B12 treatment improved insulin resistance and endothelial dysfunction in patients with metabolic syndrome [12]. Thus both homocysteine and insulin resistance have roles in the pathophysiology of hypertension [29].

The progression of prehypertension to hypertension inflammation plays a major role [30]. Cross-sectional evidence has demonstrated higher CRP levels among individuals with prehypertension [31]. Blood pressure may be increased due to the high level of CRP decreasing the nitric oxide production in endothelial cells. CRP may also function as a pro-atherosclerotic factor by up-regulating angiotensin type 1 receptor expression [32]. On treatment with high doses of water-soluble vitamins in subjects with prehypertension, there was a significant decrease in the levels of hs-CRP when compared to subjects of prehypertension on placebo. In prehypertensive subjects following a high dose of water-soluble vitamins, there was a significant decrease in hs-CRP when compared to their basal levels, but no significant change was found in the placebo group. Our results are consistent with Church et al., who conducted a six-month, randomized, double-blind, placebo-controlled trial [33]. Block et al. [34] found that supplementation of vitamin C significantly reduced the CRP level when compared to placebo in healthy non-smokers.

Limitations

The major limitations of the study are the short duration of the study and the small sample size. Further, the follow-up of the subjects with prehypertension could not be done in this study. Endothelial function and the effect of diet and exercise were not investigated among the subjects.

## Conclusions

The data from the present study concluded that supplementation with water-soluble vitamins mitigates the progression of prehypertension to hypertension by decreasing the homocysteine level, insulin resistance, and hs-CRP. Further supplementation of water-soluble vitamins might reduce the chance of CVD risk among prehypertensive individuals.
